# Plasma N-terminal pro-B-type natriuretic peptide and urinary aldosterone-to-creatinine ratio in healthy Chihuahuas

**DOI:** 10.1186/s12917-024-04344-w

**Published:** 2024-11-01

**Authors:** Alberto Galizzi, Greta Dossi, Paola Pocar, Vitaliano Borromeo, Chiara Locatelli

**Affiliations:** Department of Veterinary Medicine and Animal Science, Università degli Studi di Milano, Via dell’Università 6, 26900 Lodi, Italy

**Keywords:** Chihuahua, NT-proBNP, Aldosterone, Natriuretic peptide, Dogs

## Abstract

**Background:**

Chihuahua represents an increasingly widespread breed predisposed to cardiac disease. N-terminal pro-B-type natriuretic peptide (NT-proBNP) might be a useful point-of-care biomarker for dogs suspected of having heart disease, but breed differences have been reported. The urinary aldosterone-to-creatinine ratio (UAldo: C) appears to be a good indicator of renin-angiotensin-aldosterone system activity in dogs, but Chihuahuas showed significantly higher UAldo: C than other breeds. The objective of this study was to assess preliminary breed-specific reference intervals for NT-proBNP and UAldo: C in healthy Chihuahuas and evaluate sex differences in these parameters.

**Results:**

Forty-three healthy Chihuahuas dogs were enrolled. The median NT-proBNP was 347 (125–515) pmol/L, and the median UAldo: C was 2.59 (1.57–4.61) µg/g. The NT-proBNP reference interval was 125 (90% CI 125–125) – 2121.4 (90% CI 941.6–2248) pmol/L. 91% of the Chihuahuas were below the nonbreed-specific cut-off (900 pmol/L). The UAldo: C reference interval was 0.6 (90% CI 0.5–0.9) – 16.8 (90% CI 10.9–27.4) µg/g. No significant sex differences in NT-proBNP or UAldo: C were found.

**Conclusions:**

The median value, interindividual coefficient of variation and reference interval of NT-proBNP were in line with those reported for other small breeds. In contrast to previous studies, no sex differences in NT-proBNP were detected. As previously suggested, Chihuahuas seem to be characterized by higher values of UAldo: C than other breeds.

## Introduction

Natriuretic peptides (NPs) counterbalance the effects of the renin-angiotensin-aldosterone system (RAAS), inducing vasodilation, diuresis and natriuresis and providing antifibrotic and antihypertrophic effects [1, 2]. In human and veterinary medicine, brain natriuretic peptide and its inactive fragment N-terminal pro-B-type natriuretic peptide (NT-proBNP) are the most studied NPs in patients with cardiovascular diseases [1–3]. Compared to other NPs, they are easier to assess because of their longer half-life and greater stability [1, 4]. Brain natriuretic peptide is primarily released by ventricles after myocardial stretch and hypoxia but also under other stimuli, such as angiotensin-II and adrenergic agonists [1, 2]. Brain natriuretic peptide and NT-proBNP are recommended for prevention, initial diagnosis, and risk stratification in patients with heart failure; they are suggested as initial diagnostic tests to confirm the diagnosis of heart failure in symptomatic patients, while the monitoring of their concentrations is a useful prognostic tool and may guide further cardiological check-ups [5, 6]. In dogs, several studies have been carried out. These peptides were found to be higher in dogs with overt and occult dilated cardiomyopathy than in controls [7–10]. N-terminal pro-B-type natriuretic peptide was independently associated with survival in dogs with the occult form, although its screening utility was limited because of low sensitivity when used alone [9]. Relevant results have been reported in dogs with myxomatous mitral valve disease (MMVD). N-terminal pro-B-type natriuretic peptide was significantly greater in dogs with both symptomatic and asymptomatic MMVD than in healthy subjects, and it was found to increase with increasing disease severity [11–15]. Moreover, NT-proBNP is predictive of all-cause and cardiac mortality and is an independent risk factor for the first onset of congestive heart failure; lower levels of this peptide after treatment are associated with longer cardiac survival [11–13, 16–18]. These results suggest that NPs, in addition to standard diagnostic tests (e.g., echocardiography and thoracic radiography), might be of great aid in stratifying the risk of morbidity and mortality and in guiding therapeutic strategies in dogs with MMVD [2]. Furthermore, several studies have shown the usefulness of brain natriuretic peptide and NT-proBNP in differentiating between dogs with cardiac and noncardiac causes of respiratory signs [19–23]. However, NT-proBNP concentration requires a careful interpretation because it can also be affected by other conditions, such as systemic hypertension and renal diseases. [24, 25] Nonbreed-specific cut-offs for NT-proBNP (900 pmol/L) have been proposed by the manufacturer of the currently available test^1^ for the screening of dogs suspected of having heart disease [26]. However, significant interbreed variability has been reported, and the proposed cut-offs might not be appropriate for some breeds, as already highlighted for Labrador Retrievers and Greyhounds [27–29]. Even sex differences have been reported for this parameter [30, 31].

The assessment of RAAS activity through the measurement of its components could be of great aid in the monitoring and therapeutic management of dogs with heart disease. Aldosterone represents the last effector of the neurohormonal cascade and has acquired a relevant role in the pathophysiology of congestive heart failure, becoming a therapeutic target in dogs with symptomatic MMVD [32–34]. The urinary aldosterone-to-creatinine ratio derived from spot urine samples has been proven to correlate well with 24-h urinary aldosterone excretion [35]; thus, it can be a valid indicator of overall RAAS activity.

Chihuahua represents an increasingly widespread breed that is predisposed to MMVD; the most common causes of death in this breed are heart disease and respiratory tract disorders [36, 37]. Thus, NT-proBNP and a related breed-specific reference intervals (RIs) might be particularly useful in this breed.

In a recent study, Chihuahuas also showed a significantly higher urinary aldosterone-to-creatinine ratio (UAldo: C) than other breeds [38]. However, the number of Chihuahuas included was low, and other factors, such as sex, appeared to simultaneously affect aldosterone levels. Although markers of RAAS activity are still far from being used in the diagnostic routine for patients with MMVD, precise monitoring and therapeutic modulation of this system, titrated on a single patient, might improve disease management.

The first aim of the present study was to determine a breed-specific RIs for plasma NT-proBNP in healthy Chihuahuas using the currently available second-generation Enzyme-linked immunosorbent assay (ELISA)^1^ and to evaluate sex differences in this variable in a single-breed population. The second aim was to assess UAldo: C in a larger population of healthy Chihuahuas, to obtain a preliminary breed-specific RI and to evaluate sex differences in UAldo: C in a single-breed population.

## Materials & methods

### Animals and study timeline

The present study was part of the research project “Identification of breed-specific reference values for echocardiographic parameters and neurohormonal biomarkers in Chihuahua breed”, approved by the Animal Welfare Organisation of the Università degli Studi di Milano (OPBA_88_2021).

The study was conducted with the informed consent of the owners. The enrolled subjects were recruited from among privately owned dogs referred to the Veterinary Teaching Hospital – Università degli Studi di Milano (January-December 2021) for cardiological screening.

Each dog underwent the following procedures in this order: collection of medical history (including diet information), indirect blood pressure measurement, complete physical examination, echocardiography, blood sampling (for complete blood count, biochemistry profile and plasma NT-proBNP measurement) and collection of a free-catch urine sample (for urinalysis and urinary aldosterone determination).

### Inclusion and exclusion criteria

Chihuahuas enrolled in the present study had to be healthy and older than 12 months. Female dogs had to be neutered or in anoestrus (i.e., at least 3 months after the last oestrus and before the subsequent proestrus) to exclude any possible influence of high progesterone/oestrogen states on NT-proBNP and UAldo: C [38–44]. Subjects with any known disease, any clinical signs or who were subjected to any pharmacological treatment at the time of examinations were excluded.

A dog was considered healthy in absence of any evidence of clinically important systemic disease, if systolic blood pressure was < 160 mmHg, echocardiography was normal and complete blood count (ADVIA 120, Bayer Corporation, Tarrytown, NY, USA), biochemical analysis (BT 3500, Biotecnica Instruments SPA, Rome, Italy) and urinalysis (Combur 10 test, Roche diagnostics, Risch-Rotkreuz, Switzerland and manual refractometer model 105, Sper Scientific, Scottsdale, AZ, USA) were within the reference ranges provided by the manufacturers.

### Systemic arterial pressure and echocardiography

Indirect systemic arterial pressure measurements were carried out with a veterinary high-definition oscillatory device^2^ following published guidelines [45].

Complete echocardiography (including M-mode, B-mode and Color Doppler echocardiography) was performed on conscious dogs by two experienced echocardiographers (AG and CL) using an ultrasonographic unit (Esaote MyLabOmega) equipped with two different multifrequency phased array probes (1–9 and 2–5 MHz, respectively) following published standards [46].

All measurements were taken from at least three consecutive cardiac cycles, and the mean was recorded. The following measurements were taken from the right parasternal short-axis view: left atrium-to-aortic root ratio (LA/Ao) measured in 2D-mode using the Hansson’s method [47] and left ventricular end-diastolic diameter (LVIDD) measured in 2D-guided M-mode with the leading edge to inner edge method at the level of the papillary muscle. Normalized left ventricular end-diastolic diameter (LVIDDn) was obtained using the allometric equation, as previously described [48].

### Sample collection, storage and analysis

Blood was drawn by peripheral venepuncture at least 6 h after a meal and collected into EDTA and serum gel tubes. After centrifugation (3750 gpm for 5 min at room temperature), the serum was used for biochemical analysis (urea, creatinine, glucose, total protein, albumin, alanine aminotransferase, and alkaline phosphatase). Blood collected into EDTA tubes was used for complete blood count and then immediately centrifuged (3750 gpm for 5 min). Plasma samples were stored at -80 °C and subsequently sent at the end of the study to the IDEXX laboratory (Korwestheim, Germany) and analysed in one single batch. Plasma NT-proBNP was measured by a second-generation canine Cardiopet^®^ proBNP test (coefficients of variation intra-assay 3.9–8.9% and inter-assay 2.0–5.0%) [49].

Urine samples were collected by spontaneous micturition, and standard urinalysis was performed with dipstick chemistry tests (Combur 10 test, Roche diagnostics, Risch-Rotkreuz, Switzerland), and refractometry (manual refractometer model 105, Sper Scientific, Scottsdale, AZ, USA). The samples were then immediately centrifuged at 1250 gpm for 5 min, and the supernatant was obtained. Urinary protein and urinary creatinine were determined by a standard colorimetric assay. The residual supernatant was stored at -80 °C until urinary aldosterone analysis.

Urinary aldosterone concentrations were determined by a commercially available species-independent ELISA kit^3^, as previously described [38]. The ELISA standard curve was linear between 250 and 3.9 pg/mL. The kit was validated for urinary aldosterone measure by recovery and dilution parallelism tests. The intra-assay coefficient of variation of aldosterone assay in urine samples ranged between 8.2 and 16.6% and the inter-assay coefficient of variation between 14.2 and 21.3%. Dog urine sample were tested in duplicates by a single operator using two kits of the same manufacturing lot simultaneously.

### Statistical analysis

Statistical analysis was performed with a commercially available statistical software (IBM SPSS^®^ Statistics 28). Normality was tested using the Kolmogorov‒Smirnov test. Normally distributed variables are reported as the mean ± standard deviation, and nonnormally distributed variables are presented as the median and interquartile range. Parametric data were compared by unpaired t-test and non-parametric data were compared by unpaired Mann-Whitney test. To compare a normally distributed variable with a non-normally distributed one, unpaired Mann-Whitney test was used. The coefficient of variation (%CV) was calculated as the ratio of the standard deviation to the mean multiplied by 100. Correlations among NT-proBNP, UAldo: C and body weight were explored by the Pearson correlation coefficient (ρ), and results were interpreted as follows: ρ ≤ 0.03 weak correlation, ρ > 0.3 and ≤ 0.7 moderate correlation, ρ > 0.7 strong correlation. Influence of age on NT-proBNP and UAldo: C were not evaluated in this study due to the predominantly young age of the patients. A P value < 0.05 was considered to indicate statistical significance.

Reference intervals (2.5-97.5% of the distribution) and 90% confidence intervals (CIs) of each limit were calculated with an open-source application (Reference Value Advisor 2.1) [50]. In accordance with published guidelines [51], the RIs obtained with the robust method (on raw or Box‒Cox transformed data) were the first choice; if the robust method did not produce a valid RIs, the RIs calculated with the nonparametric method were reported. Potential outliers were identified by the same software via the combination of visual inspection, the Tukey test and the Dixon-Reed test. However, in accordance with the American Society for Veterinary Clinical Pathology guidelines [51], as all dogs included were selected randomly from a well-defined population and their health was confidently established, no outliers were removed from the analysis.

Values of NT-proBNP below the limit of detection (250 pmol/L) were reported as half of the lower limit for the statistical analysis [52, 53].

## Results

The study population consisted of 43 healthy chihuahuas. Twenty-six were intact females, 14 were intact males, and 3 were neutered males. Body condition scores were 3/9 for 1 dog, 4/9 for 18 dogs, 5/9 for 18 dogs, 6/9 for 4 dogs and 7/9 for 2 dogs. The demographic, echocardiographic and laboratory variables are reported in Table 1.

**Table 1:** Demographic, echocardiographic and laboratory variables for 43 healthy chihuahuas

	All (*n* = 43)	Females (*n* = 26)	Males (*n* = 17)	*P*
Age (years)	2.32 (1.19–4.11)	2.16 (1.17–3.86)	2.82 (2.10–5.90)	0.099
Body Weight (kg)	2.71 (2.39–3.10)	2.62 (2.35–2.84)	2.96 (2.57–3.53)	0.020*
BCS	5 (4–5)	4.75 (4–5)	5 (4-5.5)	0.411
SAP (mmHg)	137.72 ± 16.76	135.19 ± 18.17	141.59 ± 13.96	0.225
LVIDD (mm)	20.7 (19.8–21.7)	20.7 (19.7-22.35)	21 (19.8–21.3)	0.852
LVIDDn	1.51 ± 0.15	1.54 ± 0.17	1.46 ± 0.10	0.056
LA/Ao	1.38 ± 0.12	1.36 ± 0.12	1.40 ± 0.12	0.245
NT-proBNP (pmol/L)	347 (125–515)	347 (255–547)	347 (125–443)	0.408
UAldo(pg/dl)	4139 (2810–7194)	4938 (2926–7405)	3242 (2672–7146)	0.449
UAldo: C (µg/g)	2.59 (1.57–4.61)	3.08 (1.92–5.60)	1.86 (1.51–4.32)	0.124

BCS Body Condition Score (scale 1–9), SAP systolic arterial pressure, LVIDD left ventricular internal diameter in diastole (M-mode), LVIDDn left ventricular internal diameter in diastole (M-mode) normalized for body weight by allometric equation, LA/Ao left atrium-to-aortic root ratio (measured from right parasternal short-axis view), NT-proBNP N-terminal pro-B-type natriuretic peptide, UAldo urinary aldosterone, UAldo: C urinary aldosterone-to-creatinine ratio

Variables were reported as mean ± standard deviation when compared by unpaired t-test (both normally distributed variables). Variables were reported as median and interquartile range when compared by unpaired Mann-Whitney test (both non-normally distributed variables or normally distributed variable vs. non-normally distributed variable)

The Males group includes both intact and neutered males

*P < 0.05 was considered statistically significant

Body weight was significantly greater in males. No other significant differences were found between males and females.

Thirty-eight dogs were fed a normal-sodium commercial diet (0.34% [OASY One Animal Protein Adult/Small Mini Salmon; OASY, Republic of San Marino], 0.4% sodium [Royal Canin Mini Adult; Royal Canin, Aimargues, France] and 0.2% sodium [Monge All Breeds Adult Rabbit with Rice and Potatoes/Monge Grain Free – Duck with Potatoes – Mini Adult; Monge & C S.p.a, Monasterolo di Savigliano, Cuneo, Italy]); 5 dogs were fed an unspecified commercial diet. Physical examination, echocardiography, complete blood count, biochemistry profile and urinalysis were unremarkable in all dogs.

The plasma NT-proBNP and urinary aldosterone concentrations are reported in Table 1. In eleven dogs (25.6%), the NT-proBNP level was below the limit of detection (< 250 pmol/L). One NT-proBNP (2248 pmol/L) and two UAldo: C (13.93 µg/g and 23.80 µg/g, respectively) samples were detected as outliers. There were no significant differences in any of the reported parameters between females and males.

Urinary aldosterone to creatinine ratio showed a significantly moderate correlation (0.397) with body weight.

The calculated RIs for NT-proBNP and UAldo: C in healthy Chihuahuas are reported in Table 2.

**Table 2:** Calculated RIs for NT-proBNP and UAldo: C in healthy chihuahuas

	Median	Min-Max	Lower RI	90% CI	Upper RI	90% CI	%CV
NT-proBNP (pmol/L)	347	125–2248	125	125–125	2121.4	941.6–2248	115
UAldo: C (µg/g)	2.54	0.57–23.80	0.6	0.5–0.9	16.8	10.9–27.4	94

NT-proBNP N-terminal pro-B-type natriuretic peptide, UAldo: C urinary aldosterone-to-creatinine ratio, RI reference interval, CI confidence interval, %CV coefficient of variation

Coefficient of variation was calculated as the ratio of the standard deviation to the mean multiplied by 100

Median and RI for NT-proBNP was calculated via the non-parametric method

Median and RI for UAldo: C was calculated via the robust method on Box-Cox transformed data

## Discussion

N-terminal pro-B-type natriuretic peptide has been reported to have significant breed-to-breed variability, and breed-specific RIs have been recommended [27–29]. To the authors’ knowledge, a specific RIs for NT-proBNP in Chihuahuas have not yet been established [29, 30]. In the present study, Chihuahuas showed a median NT-proBNP of 347 pmol/L, with a calculated 95% RI of 125-2121.4 pmol/L. The median value was in line with or lower than those reported in other small breed dogs [29, 30]. The calculated RI was wide, but this was not an unexpected result. Rather, it likely reflects the high biological variability of NT-proBNP that has been previously documented in dogs, in the form of both intraindividual and within-group variation and presence of outlier [28–30, 54, 55]. Wide RIs were already found in healthy Labrador Retrievers (275–2100 pmol/L) [28] and in a healthy population of dogs of different small breeds (157–2842 pmol/L), where the interindividual coefficient of variation (CV) ranged from 62% in CKCSs to 100% in miniature poodles [30]. In the present study, the interindividual CV was 115%, confirming a relevant interindividual variability of NT-proBNP even within the same breed.

The interpretive criteria indicated by the manufacturer propose a cut-off of 900 pmol/L as a screening tool in dogs with suspected heart disease (presence of heart murmur or at-risk breed) [26]. 91% (39/43) of Chihuahuas enrolled in this study had an NT-proBNP concentration < 900 pmol/L; the remaining 4 dogs had values of 907, 961, 982 and 2248 pmol/L, respectively. Based on these results, the generic cut-off of 900 pmol/L appears to be suitable for this breed, although a comparison of Chihuahuas with heart disease patients would be necessary to establish an accurate breed-specific cut-off. Exceeding values could also occur in healthy Chihuahuas, likely because of the normal biological variation in NT-proBNP. This characteristic is more problematic in patients with heart diseases because it can make it difficult to interpret disease progression and response to therapy in the face of changes in NT-proBNP [55, 56]. Thus, individual serial monitoring should be performed in dogs with heart disease, and previous studies have suggested that a change of approximately 50–70% [depending on the American College of Veterinary Internal Medicine (ACVIM) stage] [33] is required to detect disease progression [55, 56].

There were no significant differences in NT-proBNP between females and males. This result is in contrast with previous reports in dogs, which found significantly higher NT-proBNP in females than in males [30, 31], and with reports in humans, where women showed significantly higher NPs concentrations than men [39, 57–59]. The mechanism underlying the influence of sex on NPs is not completely clear, but it is thought to be related to the combined effects of different sex hormones (e.g., oestrogens, progesterone and androgens) [39–44]. The different results between the present study and the previous ones [30, 31] might be related to the smaller sample size and/or more homogeneous population [i.e., single breed and restricted ranges of age and body weight (BW)]; moreover, different levels of sex hormones could have played a role since they were not measured in any of these studies, and all intact females in the present study were in anoestrus (i.e., low progesterone and oestrogens). Measuring NT-proBNP along with sex hormones in different sexes/neuters and in different phases of the reproductive cycle will help elucidate the relationship between NPs and sex.

Regarding UAldo: C, several studies have investigated it in healthy dogs, finding values < 1.0 µg/g; however, they were carried out on research dogs (hound-type dogs or beagles) and in a controlled experimental setting [60]. Ames et al. (2022) [61] reported a median value of 0.64 (0.54–0.83) µg/g and a RI of 0.23 (90% CI 0.17–0.32)-1.82 (90% CI 1.27–2.5) µg/g for 31 healthy client-owned dogs (17 spayed females and 14 neutered males; breed not specified), with a median BW of 22.2 (14.7–31.5) kg. A more recent study reported a median value of 0.47 (0.34–0.62) µg/g and a RI of 0.2 (90% CI 0.1–0.2)-1.2 (90% CI 1.0-1.3) µg/g for 60 healthy dogs with a median BW of 20.7 (8.4–29.3) kg; however, nor breed neither sex was specified [62]. The median value and the breed-specific RI found in the present study were much higher and wider. Two main differences may have contributed to this discrepancy: sex and breed.

All female dogs included were spayed in the study of Ames et al. [61], while those enrolled in the present study were intact. An author’s previous study revealed significantly higher UAldo: C in intact females than in males and spayed females [38]. Moreover, a progesterone-aldosterone relationship has already been demonstrated in the chihuahua breed [63]. Recently, Adin and Hernandez [64] reported significantly higher angiotensin-converting enzyme activity in intact females Dobermann Pinscher (not in oestrus, not pregnant or lactating) than in spayed females and intact males of the same breed. In the present study, UAldo: C was higher in intact females than in males, although the difference between groups was not statistically significant. In the authors’ previous study, the phase of the reproductive cycle was unknown [38], while in the present study, all female dogs were in anoestrus. Sex differences in RAAS activity are primarily attributed to the effect of female sex hormones [38, 63–73]. It can be presumed that high progesterone/oestrogen states (e.g., dioestrus, oestrus) enhance the discrepancy in RAAS activity between sexes/neuter status, while this difference is blunted in the case of low concentrations of these hormones (e.g., anoestrus). However, although oestradiol and progesterone concentrations in intact females-anoestrus and spayed females seem to overlap [74], the production of ovarian hormones is completely absent in spayed females and males. In contrast, ovaries are not quiescent during anoestrus, and hormone fluctuations could occur [75]; however, whether they are sufficient to cause an increase in RAAS activity compared to that of spayed females or males remains to be determined.6,438,637,365. Further investigations in the field of sex-RAAS relationships in dogs are warranted.

Breed was the other main difference between the two RIs of UAldo: C. In the studies of Ames et al. [61] and Hammond et al., [62] the breed of healthy dogs was not specified, but they were likely of medium/large size considering the reported BW [61, 62]. In the authors’ previous study, Chihuahua and Cavalier King Charles Spaniels, UAldo: C was significantly higher than other breeds [38]. In the present study, the values of UAldo: C in Chihuahua males and females appeared to be much higher than those reported for other breeds. The reason for these elevated UAldo: C values in Chihuahua remains to be determined. Polymorphisms of genes encoding RAAS components could be a possible explanation [76–78]. However, other countless and unexplored breed-specific physiological factors could have played a role, considering the relevant between-breed genetic variation reported in canine species [79]. For example, breed differences in heart rate, systemic blood pressure and catecholamine concentrations have been reported in dogs [29, 80–82].

Overall, these sex and breed differences may have led to both higher absolute values and greater interindividual variability in UAldo: C in the present study than in the studies of Ames et al. [61] and Hammond et al. [62]. Other factors, such as circadian aldosterone variations [83], dietary sodium intake [84], electrolytes and blood pressure fluctuations, hydration status and changes in sympathetic activity, may also contribute to both within- and between studies variability [61, 85]. Additional comparisons are difficult to make because, in contrast to NT-proBNP, UAldo: C does not have a standardized assay, lack of interpretative criteria and its use in patients with heart diseases is supported by many fewer studies. All these factors limit the diagnostic power of UAldo: C as a point-of-care biomarker, as well as the utility of a reference interval in a clinical setting. As previously suggested, individual longitudinal monitoring is currently preferred [38, 61].

The present study has several limitations. First, the low number of subjects may have limited the statistical power of the study; in particular, the ideal number of subjects for the calculation of a RI is ≥ 120 in veterinary medicine [51]. Second, because of the biological variability of NT-proBNP and UAldo: C [54–56, 83], multiple measurements (i.e., daily and weekly) and a more standardized urine and blood sampling time would have improved the accuracy of the results. Third, the absence of spayed females, low number of neutered males and lack of sex hormones measurements limit the consideration of the relationships between sex and both NT-proBNP and UAldo: C, and further investigations in this field are encouraged. Lastly, the influence of age on NT-proBNP could not be evaluated in this study due to the predominantly young age of the patients.

## Conclusion

The present study assessed Chihuahua-specific preliminary RIs for NT-proBNP and UAldo: C, which represent different aspects of the same neurohormonal system. The median values, RIs and interindividual CV of NT-proBNP are in line with those previously reported for other small breed dogs [29, 30]. The proposed non-breed-specific cut-off of 900 pmol/L [26] seems to be valid for most healthy Chihuahuas, although extreme values in normal subjects might occur even in this breed. In contrast to previous studies [30, 31], no sex differences in NT-proBNP were detected. As previously suggested, Chihuahuas seem to be characterized by higher values of UAldo: C than other breeds. In the current state of literature, the use of a RI for UAldo: C seems to be worthless in a clinical setting, and an individual longitudinal approach should be pursued. However, the results should be interpreted in conjunction with a physical examination and complementary tests, such as echocardiography, due to the high biological variability of biomarkers.

## Data Availability

The datasets used and/or analysed during the current study are available from the corresponding author on reasonable request.

## Declarations

### Notes

^1^ Cardiopet^®^ proBNP test, IDEXX Laboratories, Westbrook, ME.

^2^ VET-HDO ^®^- MONITOR, S + B medVET GmbH, Babenhausen, Germany.

^3^ Enzo Life Sciences Aldosterone ELISA kit, Enzo Life Sciences Inc., Farmingdale, NY, USA.

## Abbreviations

ACVIM: American College of Veterinary Internal Medicine
BW: Body Weight
CI: Confidence Interval
CIs: Confidence Intervals
CV: Coefficient of Variation
ELISA: Enzyme-Linked Immunosorbent Assay
LA/Ao: Left Atrium-to-Aortic Root Ratio
LVIDD: Left Ventricular Internal Diameter in Diastole (M-mode)
LVIDDn: Left Ventricular Internal Diameter in Diastole (M-mode) normalized for body weight by allometric equation
MMVD: Myxomatous Mitral Valve Disease
NPs: Natriuretic Peptides
NT-proBNP: N-terminal pro-B-type natriuretic peptide
RAAS: Renin-Angiotensin-Aldosterone System
RI: Reference Interval
RIs: Reference Intervals
SAP: Systolic Arterial Pressure
UAldo: Urinary Aldosterone
UAldo: C: Urinary Aldosterone-to-Creatinine Ratio

## References

[1] Kimmenade RRJ, Januzzi JL Jr. The evolution of the natriuretic peptides - current applications in human and animal medicine. J Vet Cardiol. 2009;11(suppl 1):S9–21. 19285934 10.1016/j.jvc.2009.01.001

[2] Oyama MA, Singletary GE. The use of NT-proBNP assay in the management of canine patients with heart disease. Vet Clin North Am Small Anim Pract. 2010;40(4):545–58. 20610010 10.1016/j.cvsm.2010.03.004

[3] Kuwahara K. The natriuretic peptide system in heart failure: diagnostic and therapeutic implications. Pharmacol Ther. 2021;227:107863. 33894277 10.1016/j.pharmthera.2021.107863

[4] Baba M, Yoshida K, Ieda M. Clinical applications of natriuretic peptides in heart failure and Atrial Fibrillation. Int J Mol Sci. 2019;20(11):2842. 31185605 10.3390/ijms20112824 PMC6600257

[5] Heidenreich PA, Bozkurt B, Aguilar D, et al. 2022 AHA/ACC/HFSA Guideline for the management of Heart failure: a report of the American College of Cardiology/American Heart Association Joint Committee on Clinical Practice guidelines. Circulation. 2022;145(18):e895–1302. 35363499 10.1161/CIR.0000000000001063

[6] McDonagh TA, Metra M, Adamo M, et al. 2021 ESC guidelines for the diagnosis and treatment of acute and chronic heart failure. Eur Heart J. 2021;42(36):3599–726. 34447992 10.1093/eurheartj/ehab368

[7] Oyama MA, Sisson DD, Solter PF. Prospective screening for occult cardiomyopathy in dogs by measurement of plasma atrial natriuretic peptide, B-type natriuretic peptide, and cardiac troponin-I concentrations. Am J Vet Res. 2007;68(1):42–7. 17199417 10.2460/ajvr.68.1.42

[8] Oyama MA, Fox PR, Rush JE, et al. Clinical utility of serum N-terminal pro-B-type natriuretic peptide concentration for identifying cardiac disease in dogs and assessing disease severity. J Am Vet Med Assoc. 2008;232(10):1496–503. 18479239 10.2460/javma.232.10.1496

[9] Singletary GE, Morris NA, O’Sullivan ML, et al. Prospective evaluation of NT-proBNP assay to detect Occult dilated cardiomyopathy and predict survival in Doberman Pinschers. J Vet Intern Med. 2012;26(6):1330–6. 22998090 10.1111/j.1939-1676.2012.1000.x

[10] Wess G, Butz V, Mahling M, et al. Evaluation of N-terminal pro-B-type natriuretic peptide as a diagnostic marker of various stages of cardiomyopathy in Doberman Pinschers. Am J Vet Res. 2011;72(5):642–9. 21529216 10.2460/ajvr.72.5.642

[11] Chetboul V, Serres F, Tissier R, et al. Association of Plasma N-Terminal Pro-B-Type Natriuretic Peptide Concentration with mitral regurgitation severity and outcome in dogs with asymptomatic degenerative mitral valve disease. J Vet Intern Med. 2009;23(5):984–94. 19572913 10.1111/j.1939-1676.2009.0347.x

[12] Hezzel MJ, Boswood A, Chang YM, et al. The combined prognostic potential of serum high-sensitivity Cardiac Troponin I and N-Terminal pro-B-Type natriuretic peptide concentrations in dogs with degenerative mitral valve disease. J Vet Intern Med. 2012;26(2):302–11. 22369312 10.1111/j.1939-1676.2012.00894.x

[13] Serres F, Pouchelon JL, Poujol L, et al. Plasma N-terminal pro-B-type natriuretic peptide concentration helps to predict survival in dogs with symptomatic degenerative mitral valve disease regardless of and in combination with the initial clinical status at admission. J Vet Cardiol. 2009;11(2):103–21. 19850546 10.1016/j.jvc.2009.07.001

[14] Tarnow I, Olsen LH, Kvart C et al. Predictive value of natriuretic peptides in dogs with mitral valve disease. Vet J. 2009; (2):195–201. 10.1016/j.tvjl.2007.12.026 18675567

[15] Takemura N, Toda N, Miyagawa Y, et al. Evaluation of plasma N-Terminal Pro-brain Natriuretic peptide (NT-proBNP) concentrations in dogs with mitral valve insufficiency. J Vet Med Sci. 2009;71(7):925–9. 19652480 10.1292/jvms.71.925

[16] Moonarmart W, Boswood A, Luis Fuentes V, et al. N-terminal pro B-type natriuretic peptide and left ventricular diameter independently predict mortality in dogs with mitral valve disease. J Small Anim Pract. 2010;51(2):84–96. 20070494 10.1111/j.1748-5827.2009.00889.x

[17] Reynolds CA, Brown DC, Rush JE, et al. Prediction of first onset of congestive heart failure in dogs with degenerative mitral valve disease: the PREDICT cohort study. J Vet Cardiol. 2012;14(1):193–202. 22366568 10.1016/j.jvc.2012.01.008

[18] Wolf J, Gerlach N, Weber K, et al. Lowered N-terminal pro-B-type natriuretic peptide levels in response to treatment predict survival in dogs with symptomatic mitral valve disease. J Vet Cardiol. 2012;14(3):399–408. 22858663 10.1016/j.jvc.2012.05.005

[19] Boswood A, Dukes-McEwan J, Loureiro J, et al. The diagnostic accuracy of different natriuretic peptides in the investigation of canine cardiac disease. J Small Anim Pract. 2008;49(1):26–32. 18005104 10.1111/j.1748-5827.2007.00510.x

[20] DeFrancesco T, Rush JE, Rozanski E, et al. Prospective clinical evaluation of an ELISA B-Type natriuretic peptide assay in the diagnosis of congestive heart failure in Dogs presenting with Cough or Dyspnea. J Vet Intern Med. 2007;21(2):243–50. 17427384 10.1892/0891-6640(2007)21[243:pceoae]2.0.co;2

[21] Fine DM, DeClue AM, Reinero CR. Evaluation of circulating amino terminal-pro-B-type natriuretic peptide concentration in dogs with respiratory distress attributable to congestive heart failure or primary pulmonary disease. J Am Vet Med Assoc. 2008;232(11):1674–9. 18518809 10.2460/javma.232.11.1674

[22] Prosek R, Sisson DD, Oyama MA, et al. Distinguishing Cardiac and Noncardiac Dyspnea in 48 dogs using plasma atrial natriuretic factor, B-Type natriuretic factor, Endothelin, and Cardiac Troponin-I. J Vet Intern Med. 2007;21(2):238–42. 17427383 10.1892/0891-6640(2007)21[238:dcandi]2.0.co;2

[23] Oyama MA, Rush JE, Rozanski EA, et al. Assessment of serum N-terminal pro-B-type natriuretic peptide concentration for differentiation of congestive heart failure from primary respiratory tract disease as the cause of respiratory signs in dogs. J Am Vet Med Assoc. 2009;235(11):1319–25. 19951101 10.2460/javma.235.11.1319

[24] Jang IS, Yoon WK, Choi EW. N-terminal pro-B-type natriuretic peptide levels in normotensive and hypertensive dogs with myxomatous mitral valve disease stage B. Ir Vet J. 2023;76(1):3. 36755290 10.1186/s13620-023-00233-0 PMC9906826

[25] Schmidt MK, Reynolds CA, Estrada AH, et al. Effect of azotemia on serum N-terminal proBNP concentration in dogs with normal cardiac function: a pilot study. J Vet Cardiol. 2009;11(Suppl 1):S81–6. 19394913 10.1016/j.jvc.2009.02.001

[26] IDEXX Laboratories interpretative criteria. 2013. https://www.idexx.com/media/filer_public/26/89/26893bee-e598-479f-ba85-b0084b2ff542/cardiopet-interpretive-criteria-canine.pdfa. Accessed 22 Feb 2023.

[27] Couto KM, Iazbik MC, Marin LM, et al. Plasma N-terminal pro-B-type natriuretic peptide concentration in healthy retired racing greyhounds. Vet Clin Pathol. 2015;44(3):405–9. 25982692 10.1111/vcp.12266

[28] Gomart S, Allaway D, Harrison M, et al. Long-term biological variability and the generation of a new reference interval for plasma N-terminal pro-B-type natriuretic peptide in Labrador retrievers. J Small Anim Pract. 2020;61(6):368–73. 32297329 10.1111/jsap.13136

[29] Sjöstrand K, Wess G, Ljungvall I, et al. Breed differences in Natriuretic Peptides in Healthy Dogs. J Vet Intern Med. 2014;28(2):451–7. 24495256 10.1111/jvim.12310 PMC4857989

[30] Misbach C, Chetboul V, Concordet D, et al. Basal plasma concentrations of N-terminal pro-B-type natriuretic peptide in clinically healthy adult small size dogs: Effect of body weight, age, gender and breed, and reference intervals. Res Vet Sci. 2013;95(3):879–85. 23993661 10.1016/j.rvsc.2013.07.025

[31] Wolf J, Gerlach N, Weber K, et al. The diagnostic relevance of NT-proBNP and proANP 31–67 measurements in staging of myxomatous mitral valve disease in dogs. Vet Clin Pathol. 2013;42(2):196–206. 23614733 10.1111/vcp.12044

[32] Bernay F, Bland JM, Hӓggström J, et al. Efficacy of spironolactone on survival in dogs with naturally occurring mitral regurgitation caused by myxomatous mitral valve disease. J Vet Intern Med. 2010;24(2):331–41. 20102506 10.1111/j.1939-1676.2009.0467.x

[33] Keene BW, Atkins CE, Bonagura JD, et al. ACVIM consensus guidelines for the diagnosis and treatment of myxomatous mitral valve disease in dogs. J Vet Intern Med. 2019;33(3):1127–40. 30974015 10.1111/jvim.15488 PMC6524084

[34] Coffman M, Guillot E, Blondel T, et al. Clinical efficacy of a benazepril and spironolactone combination in dogs with congestive heart failure due to myxomatous mitral valve disease: the BEnazepril Spironolactone STudy (BESST). J Vet Intern Med. 2021;35(4):1673–87. 34028078 10.1111/jvim.16155 PMC8295662

[35] Gardner SY, Atkins CE, Rausch WP, et al. Estimation of 24-h aldosterone secretion in the dog using the urine aldosterone:creatinine ratio. J Vet Cardiol. 2007;9(1):1–7. 17689463 10.1016/j.jvc.2006.11.001

[36] Mattin MJ, Boswood A, Church DB, et al. Prevalence of and risk factors for degenerative mitral valve disease in dogs attending primary-care veterinary practices in England. J Vet Intern Med. 2015;29(3):847–54. 25857638 10.1111/jvim.12591 PMC4895395

[37] O’Neill DG, Packer RMA, Lobb M, et al. Demography and commonly recorded clinical conditions of chihuahuas under primary veterinary care in the UK in 2016. BMC Vet Res. 2020;16(1):42. 32046714 10.1186/s12917-020-2258-1 PMC7014602

[38] Galizzi A, Bagardi M, Stranieri A, et al. Factors affecting the urinary aldosterone-to-creatinine ratio in healthy dogs and dogs with naturally occurring myxomatous mitral valve disease. BMC Vet Res. 2021;17(1):15. 33413406 10.1186/s12917-020-02716-6 PMC7792040

[39] Lam CSP, Cheng S, Choong K, et al. Influence of sex and hormone status on circulating natriuretic peptides. J Am Coll Cardiol. 2011;58(6):618–26. 21798425 10.1016/j.jacc.2011.03.042 PMC3170816

[40] Chang AY, Abdullah SM, Jain T, et al. Associations among androgens, estrogens, and natriuretic peptides in young women: observations from the Dallas Heart Study. J Am Coll Cardiol. 2007;49(1):109–16. 17207730 10.1016/j.jacc.2006.10.040

[41] Hong M, Yan Q, Tao B, et al. Estradiol, progesterone and testosterone exposures affect the atrial natriuretic peptide gene expression in vivo in rats. Biol Chem Hoppe Seyler. 1992;373(4):213–8. 1534483 10.1515/bchm3.1992.373.1.213

[42] Maffei S, Clerico A, Iervasi G, et al. Circulating levels of cardiac natriuretic hormones measured in women during menstrual cycle. J Endocrinol Invest. 1999;22(1):1–5. 10090129 10.1007/BF03345470

[43] Maffei S, Del Ry S, Prontera C, et al. Increase in circulating levels of cardiac natriuretic peptides after hormone replacement therapy in postmenopausal women. Clin Sci (Lond). 2001;101(5):447–53. 11672449

[44] Yeko TR, Rao PS, Parsons AK, et al. Atrial natriuretic peptide, oestradiol and progesterone in women undergoing spontaneous and gonadotrophin-stimulated ovulatory cycles. Hum Reprod. 1995;10(11):2872–4. 8747035 10.1093/oxfordjournals.humrep.a135810

[45] Acierno MJ, Brown S, Coleman AE, et al. ACVIM consensus statement: guidelines for the identification, evaluation, and management of systemic hypertension in dogs and cats. J Vet Intern Med. 2018;32:1803–22. 30353952 10.1111/jvim.15331 PMC6271319

[46] Thomas WP, Gaber CE, Jacobs GJ, et al. Recommendations for standards in transthoracic two-dimensional echocardiography in the dog and cat. Echocardiography committee of the specialty of cardiology, American college of veterinary internal medicine. J Vet Intern Med. 1993;7:247–52. 8246215 10.1111/j.1939-1676.1993.tb01015.x

[47] Hansson K, Häggström J, Kvart C, et al. Left atrial to aortic root indices using two-dimensional and m-mode echocardiography in cavalier king charles spaniels with and without left atrial enlargement. Vet Radiol Ultrasound. 2002;43:568–75. 12502113 10.1111/j.1740-8261.2002.tb01051.x

[48] Cornell CC, Kittleson MD, Della Torre P, et al. Allometric scaling of Mmode cardiac measurements in normal adult dogs. J Vet Intern Med. 2004;18:311–21. 15188817 10.1892/0891-6640(2004)18<311:asomcm>2.0.co;2

[49] Cahill RJ, Pigeon K, Strong-Townsend MI, et al. Analytical validation of a second-generation immunoassay for the quantification of N-terminal pro-B-type natriuretic peptide in canine blood. J Vet Diagn Invest. 2015;27(1):61–7. 25525139 10.1177/1040638714562826

[50] Geffré A, Concordet D, Braun JP, et al. Reference Value Advisor: a new freeware set of macroinstructions to calculate reference intervals with Microsoft Excel. Vet Clin Pathol. 2011;40(1):107–12. 21366659 10.1111/j.1939-165X.2011.00287.x

[51] Friedrichs KR, Harr KE, Freeman KP, et al. ASVCP reference interval guidelines: determination of de novo reference intervals in veterinary species and other related topics. Vet Clin Pathol. 2021;41(4):441–53. 10.1111/vcp.12006 23240820

[52] Keizer RJ, Jansen RS, Rosing H, et al. Incorporation of concentration data below the limit of quantification in population pharmacokinetic analyses. Pharmacol Res Perspect. 2015;3:1–15. 10.1002/prp2.131 PMC4448983 26038706

[53] Ogden TL. Handling results below the level of detection. Ann Occup Hyg. 2010;54(3):255–6. 20067938 10.1093/annhyg/mep099

[54] Kellihan HB, Oyama MA, Reynolds CA, et al. Weekly variability of plasma and serum NT-proBNP measurements in normal dogs. J Vet Cardiol. 2009;11(suppl 1):S93–7. 19395335 10.1016/j.jvc.2009.03.003

[55] Winter RL, Saunders AB, Gordon SG, et al. Biologic variability of N-terminal pro-brain natriuretic peptide in healthy dogs and dogs with myxomatous mitral valve disease. J Vet Cardiol. 2017;19(2):124–31. 28111138 10.1016/j.jvc.2016.11.001

[56] Ruaux C, Scollan K, Suchodolski JS, et al. Biologic variability in NT-proBNP and cardiac troponin-I in healthy dogs and dogs with mitral valve degeneration. Vet Clin Pathol. 2015;44(3):420–30. 26108974 10.1111/vcp.12268

[57] Fragopoulu E, Panagiotakos DB, Pitsavos C, et al. N-terminal ProBNP distribution and correlations with biological characteristics in apparently healthy Greek population: ATTICA study. Angiology. 2010;61(4):397–404. 19815605 10.1177/0003319709350134

[58] Loke I, Squire IB, Davies JE, et al. Reference ranges for natriuretic peptides for diagnostic use are dependent on age, gender and heart rate. Eur J Heart Fail. 2003;5(5):599–606. 14607197 10.1016/s1388-9842(03)00108-9

[59] Redfield MM, Rodeheffer RJ, Jacobsen SJ, et al. Plasma brain natriuretic peptide concentration: impact of age and gender. J Am Coll Cardiol. 2002;40(5):976–82. 12225726 10.1016/s0735-1097(02)02059-4

[60] Ames MK, Atkins CE, Lantis AC, et al. Evaluation of subacute change in RAAS activity (as indicated by urinary aldosterone:creatinine, after pharmacologic provocation) and the response to ACE inhibition. J Renin Angiotensin Aldosteron Syst. 2016;17(1):1470320316633897. 10.1177/1470320316633897 PMC5843907 27009288

[61] Ames MK, Vaden SL, Atkins CE, et al. Prevalence of aldosterone breakthrough in dogs receiving renin-angiotensin system inhibitors for proteinuric chronic kidney disease. J Vet Intern Med. 2022;36(6):2088–97. 36350258 10.1111/jvim.16573 PMC9708418

[62] Hammond HH, Ames MK, Domenig O, et al. The classical and alternative circulating renin-angiotensin system in normal dogs and dogs with stage B1 and B2 myxomatous mitral valve disease. J Vet Intern Med. 2023;37(3):875–86. 36951394 10.1111/jvim.16687 PMC10229370

[63] Galizzi A, Dossi G, Borromeo V, et al. Aldosterone-progesterone relationship in sexually intact Chihuahua bitches. BMC Vet Res. 2023;19(1):144. Sep 5. 37670293 10.1186/s12917-023-03704-2 PMC10478304

[64] Adin DB, Hernandez JA. Influence of sex on renin-angiotensin-aldosterone system metabolites and enzymes in Doberman Pinschers. J Vet Intern Med. 2023;37(1):22–7. 36412252 10.1111/jvim.16589 PMC9889697

[65] Braley LM, Menachery AI, Yao, et al. Effect of progesterone on aldosterone secretion in rats. Endocrinology. 1996;137:4773–8. 8895346 10.1210/endo.137.11.8895346

[66] Chidambaram M, Duncan JA, Lai VS, et al. Variation in the renin angiotensin system through the normal menstrual cycle. J Am Soc Nephrol. 2002;13:446–52. 11805174 10.1681/ASN.V132446

[67] Hirshoren N, Tzoran I, Makrienko I, et al. Menstrual cycle effects on the neurohumoral and autonomic nervous systems regulating the cardiovascular system. J Clin Endocrinol Metab. 2002;87:1569–75. 11932284 10.1210/jcem.87.4.8406

[68] Komukai K, Mochizuki S, Yoshimura M. Gender and the renin-angiotensin aldosterone system. Fundam Clin Pharmacol. 2010;24:687–9. 20608988 10.1111/j.1472-8206.2010.00854.x

[69] Mihailidou AS, Ashton AW. Cardiac effects of aldosterone: does gender matter? Steroids. 2014;91:32–7. 25173820 10.1016/j.steroids.2014.08.013

[70] O’Donnell E, Floras JS, Harvey PJ. Estrogen status and the renin angiotensin aldosterone system. Am J Physiol Regul Integr Comp Physiol. 2014;307:498–500. 10.1152/ajpregu.00182.2014 24944241

[71] Oelkers WK. Effects of estrogens and progestogens on the renin-aldosterone system and blood pressure. Steroids. 1996;61(4):166–71. 8732994 10.1016/0039-128x(96)00007-4

[72] Johnson JA, Davis JO, Baumber JS, et al. Effects of estrogens and progesterone on electrolyte balances in normal dogs. Am J Physiol. 1970;219(6):1691–7. 5485687 10.1152/ajplegacy.1970.219.6.1691

[73] Szmuilowicz ED, Adler GK, Williams JS, et al. Relationship between aldosterone and progesterone in the human menstrual cycle. J Clin Endocrinol Metab. 2006;91:3981–7. 16868049 10.1210/jc.2006-1154

[74] Buijtels JJ, Beijerink NJ, Kooistra HS, et al. Effects of gonadotrophin releasing hormone administration on the pituitary-ovarian axis in anoestrous vs ovariectomized bitches. Reprod Domest Anim. 2006;41(6):555–61. 17107517 10.1111/j.1439-0531.2006.00714.x

[75] Olson PN, Bowen RA, Behrendt MD, et al. Concentrations of Reproductive hormones in Canine serum throughout late Anestrus, Proestrus and Estrus. Biol Reprod. 1982;27(5):1196–206. 6819009 10.1095/biolreprod27.5.1196

[76] Adin DB, Atkins CE, Domenig O, et al. Renin-angiotensin aldosterone profile before and after angiotensin-converting enzyme-inhibitor administration in dogs with angiotensin-converting enzyme gene polymorphism. J Vet Intern Med. 2020;34:600–6. 32112596 10.1111/jvim.15746 PMC7097578

[77] Adin DB, Atkins CE, Friedenberg SG, et al. Prevalence of an angiotensin-converting enzyme gene variant in dogs. Canine Med Genet. 2021;8:1–6. 34256860 10.1186/s40575-021-00105-2 PMC8276509

[78] Meurs KM, Stern JA, Atkins CE, et al. Angiotensin-converting enzyme activity and inhibition in dogs with cardiac disease and an angiotensin-converting enzyme polymorphism. J Renin Angiotensin Aldosterone Syst. 2017;18:1470320317737184. 29069972 10.1177/1470320317737184 PMC5843865

[79] Parker HG, Ostrander EA. Canine genomics and genetics: running with the pack. PLoS Genet. 2005;1(5):e58. 16311623 10.1371/journal.pgen.0010058 PMC1287952

[80] Bodey AR, Michell AR. Epidemiological study of blood pressure in domestic dogs. J Small Anim Pract. 1996;37:116–25. 8683954 10.1111/j.1748-5827.1996.tb02358.x

[81] Höglund K, Hanås S, Carnabuci C, et al. Blood pressure, heart rate, and urinary catecholamines in healthy dogs subjected to different clinical settings. J Vet Intern Med. 2012;26:1300–8. 22998326 10.1111/j.1939-1676.2012.00999.x

[82] Rasmussen CE, Vesterholm S, Ludvigsen TP, et al. Holter monitoring in clinically healthy Cavalier King Charles Spaniels, Wire-haired dachshunds, and Cairn Terriers. J Vet Intern Med. 2011;25:460–8. 21418322 10.1111/j.1939-1676.2011.0707.x

[83] Mochel JP, Fink M, Peyrou M, et al. Chronobiology of the renin-angiotensin-aldosterone system in dogs: relation to blood pressure and renal physiology. Chronobiol Int. 2013;30(9):1144–59. 23931032 10.3109/07420528.2013.807275

[84] Lovern CS, Swecker WS, Lee JC, et al. Additive effects of a sodium chloride restricted diet and furosemide administration in healthy dogs. Am J Vet Res. 2001;62(11):1793–6. 11703026 10.2460/ajvr.2001.62.1793

[85] Ames MK, Atkins CE, Pitt B. The renin-angiotensin-aldosterone system and its suppression. J Vet Intern Med. 2019;33(2):363–82. 30806496 10.1111/jvim.15454 PMC6430926

